# Sleep disturbances and disorders in the memory clinic: Self-report, actigraphy, and polysomnography

**DOI:** 10.1177/13872877251338065

**Published:** 2025-05-05

**Authors:** Aaron Lam, Dexiao Kong, Angela L D’Rozario, Catriona Ireland, Rebekah M Ahmed, Zoe Menczel Schrire, Loren Mowszowski, Johannes Michaelian, Ron R Grunstein, Sharon L Naismith

**Affiliations:** 1 Healthy Brain Ageing Program, The Brain and Mind Centre, University of Sydney, Camperdown, NSW, Australia; 2 School of Psychology, Faculty of Science, University of Sydney, Camperdown, NSW, Australia; 3 Woolcock Institute of Medical Research, Macquarie University, Macquarie Park, NSW, Australia; 4 CogSleep, NHMRC Centre of Research Excellence, Camperdown, NSW, Australia; 5 Department of Respiratory and Sleep Medicine, Royal Prince Alfred Hospital, Camperdown, NSW, Australia; 6 Faculty of Medicine and Health, University of Sydney, Camperdown, NSW, Australia; 7 Charles Perkins Centre, University of Sydney, Camperdown, NSW, Australia

**Keywords:** aging, Alzheimer's disease, circadian rhythm, dementia, mild cognitive impairment, REM sleep behavior disorder, sleep apnea

## Abstract

**Background:**

Sleep disturbances are common in dementia but rarely studied in memory clinics.

**Objective:**

In a memory clinic setting we aimed to (1) identify rates of obstructive sleep apnea (OSA), abnormal sleep duration, circadian phase shift, insomnia, poor sleep quality, and REM sleep behavior disorder (RBD); (2) assess concordance between self-reported and actigraphy-derived measures; investigate associations between sleep disturbances; and (3) neuropsychological performance and (4) cognitive status.

**Methods:**

Adults over 50 at a memory clinic between 2009–2024 were included. OSA was assessed via polysomnography and prior history. Sleep duration and circadian phase were measured by self-report and actigraphy. Self-report questionnaires evaluated insomnia, sleep quality, and RBD. Global cognition, processing speed, memory, and executive function were assessed. Analysis of Covariance and multinomial logistic regression examined the impact of OSA, sleep duration, insomnia, and sleep quality on cognition and cognitive status.

**Results:**

1234 participants (Mage 67.2, 46%M) were included. 75.3% had OSA, while 12.7% were previously diagnosed. Insomnia affected 12.0%, 54.3% had poor sleep quality, and 14.2% endorsed RBD symptoms. Self-reported short (30.5%) and long (10.2%) sleep exceeded actigraphy rates (8.5% and 5.1%) with poor concordance between measures. OSA was linked to impaired global cognition and memory (p < 0.05). Prolonged sleep predicted deficits in global cognition, processing speed, memory, and executive function and a higher risk of aMCI (all p < 0.05). Poor sleep quality was linked to better memory (p < 0.05).

**Conclusions:**

Despite discrepancies between self-reported and objective prevalence rates, sleep disturbances are highly prevalent in memory clinics and impact cognition, necessitating further examination.

## Introduction

Sleep disturbances are common in older adults and can include obstructive sleep apnea (OSA), short or long sleep duration, insomnia, rapid eye movement (REM) sleep behavior disorder (RBD), alterations in sleep phase or timing, as well as reports of poorer sleep quality compared to younger adults.^1^ Epidemiological studies show that up to 83% of males and 60% of females between 40 and 85 years meet the criteria for at least mild OSA.^2^ Regarding sleep duration, up to 28.3% of older adults sleep less than six hours.^3^ The overall prevalence of insomnia symptoms is up to 48% in older adults.^4^ An epidemiological survey of 5074 older adults found that 18% reported poor sleep quality.^5^ RBD is a parasomnia where abnormal dream enactment is present during REM sleep. Although the prevalence of RBD is low, being around 2%^6^ in community samples, it is highly predictive of Parkinson's disease, dementia with Lewy bodies, and multiple system atrophy, with 73.5% of older adults with RBD converting to one of these neurodegenerative syndromes after 12 years of follow-up.^7^

A converging body of work suggests that various aspects of sleep disturbance may contribute to the risk of cognitive decline and dementia.^8^ A meta-analysis of self-reported sleep quality, duration, and cognitive function in 97,264 older adults demonstrated that short sleep (five hours or less) and long sleep (nine hours or more) were associated with impairment in the cognitive domains of working memory, verbal memory and executive function.^9^ Both self-reported and clinically diagnosed insomnia have been associated with cognitive decline and dementia risk.^10^ Besides quantity of sleep, quality of sleep has been identified as a potential contributor to dementia. Indeed, self-reported poor sleep quality has been linked to poorer global cognition and dementia risk.^11^ Additionally, OSA has been linked with brain atrophy,^12^ white matter change, increased levels of amyloid-β,^13^ impaired sleep-dependent memory,^14^ increased risk of cognitive decline,^15–18^ and dementia.^19^ Notably, these associations have primarily been reported in distinct sampling frames, including middle-aged adults from sleep clinics or older community samples.^17,20^

Currently, there are only a few reports examining rates and correlates of sleep disorders in settings where people are primarily presenting with cognitive complaints, such as in memory and cognition clinics. Around one-third of attendees may meet the criteria for mild cognitive impairment (MCI), a ‘high risk’ state, whereby 45% will progress to dementia within five years.^21^ Some may even present with subjective cognitive impairment (SCI) and could be in a preclinical phase of dementia. Those with SCI are twice as likely to convert to MCI and dementia than those without.^22,23^ Since pathological changes leading to dementia occur up to 10–20 years before clinical symptoms emerge,^24^ understanding rates and optimal detection methods for sleep disturbance in these at-risk populations is warranted, not only to guide treatment approaches that may improve functioning in the short-term, but also to inform potential secondary prevention strategies.

While preliminary evidence suggests that rates of sleep disturbances may be higher in specialist memory clinics when compared to community samples of older people,^25,26^ they are not yet routinely assessed in this health setting.^27^ This could be attributed to various factors, including time constraints, a core focus on the presenting cognitive problem (and lack of attention to dementia risk reduction or strategies to target modifiable or contributing factors), lack of clarity and clinician knowledge or skills regarding the heterogeneous nature of sleep disturbances, lack of accessibility to sleep expertise and related tools such as actigraphy, lack of treatment options, or long wait times, or inaccessibility of formal polysomnography (PSG).^21^ While self-report measures may offer an easy alternative to objective measures, multiple questionnaires exist for the various aspects of sleep. Most have not been validated for use in cognitively impaired individuals. Furthermore, even in cognitively intact older adults, these questionnaires have performed poorly against objective sleep measures.^28^

The current study is a first step in addressing these gaps and has three key aims. First, we sought to determine rates of various sleep disturbances (OSA, short and long sleep duration, insomnia, sleep quality, advanced and delayed sleep phase, and RBD) in a specialist early intervention memory clinic, specifically in those with SCI, MCI, and early dementia. Second, we explored the agreement between self-report sleep and actigraphy-derived sleep measures to identify potential discrepancies that could impact the accuracy and reliability of sleep assessments in clinical and research settings. Finally, we set out to investigate the relationship between OSA, sleep duration, insomnia, and sleep quality and neuropsychological performance in those with SCI and MCI. We hypothesized that sleep disturbances and poor sleep quality would be linked to worse neuropsychological performance. Lastly, we explored whether these sleep disturbances would predict cognitive classifications (SCI, naMCI, and aMCI).

## Methods

### Participants

We recruited consecutive individuals from the Healthy Brain Ageing Clinic, Sydney, Australia between 2009 and 2024. As previously described,^29^ this specialist early intervention memory clinic provides comprehensive medical, neuropsychological, and mood assessments for older adults. Inclusion criteria are: age ≥50 years; concerns about new-onset cognitive decline; and referral by a general practitioner or medical specialist. Exclusion criteria are: a pre-existing diagnosis of dementia or a Mini-Mental State Examination (MMSE) score <20; neurological, non-affective psychiatric or other medical conditions known to affect cognition; head injury with loss of consciousness > 30 min; current significant alcohol misuse (>14 standard drinks per week); intellectual disability; history of stroke; and inadequate English to complete neuropsychological assessment. The study was approved by the University of Sydney Human Research Ethics Committee (No. 2019/271 and No. 2012/1873) and all participants provided written informed consent before any study procedures were conducted.

### Medical assessment

Using a semi-structured interview, a medical specialist (geriatrician or neurologist) conducted a physical examination, medical and sleep disorder history, anthropometrics for calculating body mass index (BMI) and recorded medication and alcohol use. The Cumulative Illness Rating Scale – Geriatric Version (CIRS-G) was administered and measured the burden of medical diseases.^30^ Rates of self-reported OSA and other sleep disorders were obtained from referrals, the medical interview, and participants were asked about previous diagnoses of sleep apnea and/or use of continuous positive airway pressure (CPAP) treatment.

### Neuropsychological and psychological assessment

A research psychologist assessed for lifetime and current Major Depression diagnoses using the Structured Clinical Interview for DSM-IV-TR^31^ or the Mini-International Neuropsychiatric Interview Version 6.0 Module A (Major Depressive Episode).^32^ Current Major Depressive episodes are reported here for descriptive purposes.

As previously described,^29^ a standardized cognitive test battery was conducted by a Clinical Neuropsychologist. It included tests of verbal learning and memory, processing speed, working memory, visual learning, language, visuospatial and aspects of executive functions. Based on prior smaller studies in cognitively intact older adults suggesting sleep disturbances may be associated with impairments in global cognition,^8^ processing speed,^33^ verbal memory,^33^ and executive functioning,^33^ we selected tests targeting these cognitive domains for the present study:

- Global cognition – The MMSE^34^ measured global cognition, ranging from 0 to 30, with higher scores indicative of better cognitive function.
- Processing speed – The Trail Making Test (TMT)^35^ comprised two parts (A and B). TMT-A assessed visuomotor speed by asking participants to draw a line between consecutively numbered circles, which was used to measure processing speed. Time to complete task (seconds) was used to measure processing speed, where higher scores indicated slower processing speed.
- Verbal Memory – The Rey Auditory Verbal Learning Test (RAVLT)^36^ is a 15-item list learning task that comprises five learning trials (RAVLT 1-5) followed by an interference trial and a 20-min delayed recall trial (RAVLT 7). RAVLT 7 score was used (/15), where higher scores (more words recalled) indicated better verbal memory.
- Executive functioning – The Trail Making Test (TMT)^35^ Part B (TMT-B) assesses set-shifting and mental flexibility by asking participants to draw a line between circles while alternating between numbers and letters. Time to task completion (seconds) was used to measure executive function performance, where higher scores indicated poorer executive function.

For descriptive purposes, premorbid general intellectual functioning was estimated using the Wechsler Test of Adult Reading.^37^

### SCI, MCI, and dementia classifications

Diagnoses and cognitive classifications were conducted via the consensus of two neuropsychologists and one medical specialist. Based on all clinical information and the full battery of neuropsychological testing (considering age and education-adjusted normative data and premorbid estimates), participants were classified as meeting clinical criteria for SCI, MCI or dementia using standardized criteria (for MCI, see Winblad et al., 2004;^38^ for SCI, see Jessen et al., 2014;^39^ for dementia, see DSM-V^40^ and Jack et al. 2011.^41^ In brief, for MCI, the presence of objective decrements of ≥1.5 standard deviations were required alongside self- or informant-based complaints of cognitive change, and at most, minimal functional change. If decrements were observed in the memory domain, they were classified as amnestic (aMCI), and otherwise as non-amnestic (naMCI). Participants who self-reported cognitive impairment but did not meet the criteria for MCI were classified as SCI. Dementia was diagnosed according to the presence of objective decrements of ≥1.5 standard deviations and the presence of functional decline according to clinical criteria (e.g., functional impairment to daily living).^40^

### Polysomnography (PSG)

As an optional component, participants were referred for a full PSG at the Woolcock Institute of Medical Research. Using Compumedics, Alice, Sandman, or REMLogic sleep systems, the PSG collected electroencephalography (EEG) activity with SINBAR setup according to the International 10–20 System of Electrode Placement and left lateral, right lateral, and right supraocular electrooculogram (EOG), electromyogram, nasal cannula, limb movement sensors, plethysmography, lead II electrocardiography, and pulse oximetry. Signals were sampled at 200 Hz (Alice) or 256 Hz (Compumedics, Sandman, and REMLogic), with a high-pass and low-pass filter at 0.3 and 50 Hz, respectively. Sleep staging and event scoring was performed using the American Academy of Sleep Medicine (AASM) v2.2 criteria by a trained sleep technician. Participants were classified as meeting the criteria for OSA if they had an apnea-hypopnea index (AHI) of five or more. Participants with OSA were further classified as mild (AHI 5-14), moderate (AHI 15-29), and severe (AHI ≥30) OSA.

### Self-report

- *Depressive symptoms* were measured by self-report using the 15-item Geriatric Depression Scale (GDS-15),^42^ where higher scores indicate greater depressive symptoms.
- *Quality of life* was measured by the first question of the World Health Organization Quality of Life (WHOQOL),^43^ which asks participants to self-report their perception of their overall quality of life on a scale from 1 to 5, where 1 specified very poor quality of life and 5 specified very good quality of life.
- *Insomnia severity* was measured by the Insomnia Severity Index (ISI).^44^ The ISI captures the subjective severity of sleep onset and maintenance, interference with daily functioning, and impairment or distress associated with sleep problems. Scores ranged from 0 to 28, where a higher score indicates greater insomnia severity. An ISI score of 0–7 suggests absence of insomnia, 8–14 suggests subthreshold (mild) insomnia, 15–21 suggests moderate insomnia, and >21 indicates severe insomnia.
- *Subjective sleep duration* and *quality* were measured with the PSQI^45^ questionnaire.
  The PSQI measures seven components of sleep, including sleep duration, sleep disturbance, sleep latency, daytime dysfunction due to sleepiness, sleep efficiency, overall sleep quality, and sleep medication use. Each component is summed to give a global score between 0 and 21, and higher scores indicate worse subjective sleep quality. Participants were classified as short or long sleepers if they, on average, slept <6 h or >9 h across all nights, respectively. A global PSQI score of 0–5 indicates good sleep quality, whilst a score ≥6 indicates poor sleep quality.
- *Advanced* and *Delayed sleep phase* was defined as at least 1.5 standard deviations from previously published mean sleep onset and offset times for older adults aged between 60–75.^46^ Participants were classified as having an advanced sleep phase if sleep onset was before 8:53pm and/or offset was before 4:53am. Delayed sleep phase was defined as sleep onset after 12:49am and/or sleep offset after 8:11am. Participants that met both criteria were considered normal sleep phase as this indicated short sleep duration rather than a change in sleep phase.
- *RBD symptoms* (e.g., dream enactment, very vivid dreams) were captured via the REM Sleep Behavior Disorder Screening Questionnaire.^47^ Scores range from 0 to 13, where higher scores indicate a greater risk of having RBD. A score of ≥5 is often used to determine RBD, with a sensitivity of 96% and specificity of 56%.^47^

### Actigraphy

As previously reported,^48^ participants were asked to wear an actigraphy watch (Actiwatch Spectrum, Minimitter-Respironics, OR) on their nondominant hand for 14 days and to complete a sleep diary that captured self-report sleep onset and offset and number of awakenings during the night. Actigraphy data were analyzed using Actiware 5.0 software. The recordings were in 30-s epochs. Sleep onset and offset were manually scored using the actigraphy-derived activity and light levels, coupled with information from the sleep diary.

Sleep onset was determined by at least three epochs of zero activity, a reduction in light, and self-reported sleep onset. Sleep offset was determined by the presence of an increase in activity from zero for at least three consecutive epochs, an increase in light, and self-reported sleep offset. Total sleep time was determined by the time between sleep onset and offset minus wake after sleep onset. For advanced and delayed sleep phase, the same criteria as self-report were applied (see above). The wake threshold value was fixed at 40 counts per epoch. Participants who had less than seven days of data or less than 60 min of sleep were excluded from this analysis. Participants were classified as short or long sleepers if they on average slept <6 h or >9 h across all nights, respectively.

### Statistical analyses

All statistical analyses were performed in SPSS v26^49^ and histograms were created using ‘ggplot2’ in R version 4.2. Percentages were calculated for rates of each category of sleep disturbance (OSA, short and long sleepers, advanced and delayed sleep phase, insomnia, poor sleepers, RBD) divided by the total number of participants, multiplied by 100.

The concordance between self-reported and actigraphy-derived rates of short and long sleep duration, and advanced and delayed sleep phases was assessed using Cohen's Kappa. An alpha level of 0.05 was set for statistical significance and a Cohen's Kappa value of 0.6 was established as the cut-off for acceptable agreement.^50^

Group differences in MMSE, processing speed, verbal memory, and executive function were examined using analysis of covariance test between the following groups: moderate and severe OSA versus without and mild OSA; short versus normal versus long sleepers; insomnia versus no insomnia; and finally, good sleepers *vs*. poor sleepers. Group analyses were initially adjusted for age, sex, and years of education. For analyses where significant group differences were found, further adjustments were made to control for depressive symptoms and the burden of medical diseases to account for potential confounding effects. Cohen's *d* was used to measure the effect size of the group differences, where 0.2 indicated a small, 0.5 indicated a moderate, and 0.8 indicated a large effect size. A sensitivity analysis was conducted to investigate whether the use of antidepressants and psychotropics influenced the relationship between sleep and cognition. Additionally, two further sensitivity analyses were performed to explore potential sex differences in this relationship. Multiple comparison corrections were applied using a family-wise error rate (FWER) adjustment, specifically the Bonferroni method, with the significance level set to 0.25 to account for the exploratory nature of these analyses.

Lastly, multinomial logistic regression was conducted to explore whether these sleep disturbances predicted cognitive impairment status (no MCI compared to naMCI or aMCI). Due to the number of sleep-related variables assessed, we applied a correction for multiple comparisons using a FWER adjustment (the Bonferroni method) to control for Type 1 error, the adjusted significance level was 0.01. Additionally, we adjusted for age, sex, education level, depressive symptoms, and burden of medical diseases.

## Results

**Figure 1.**
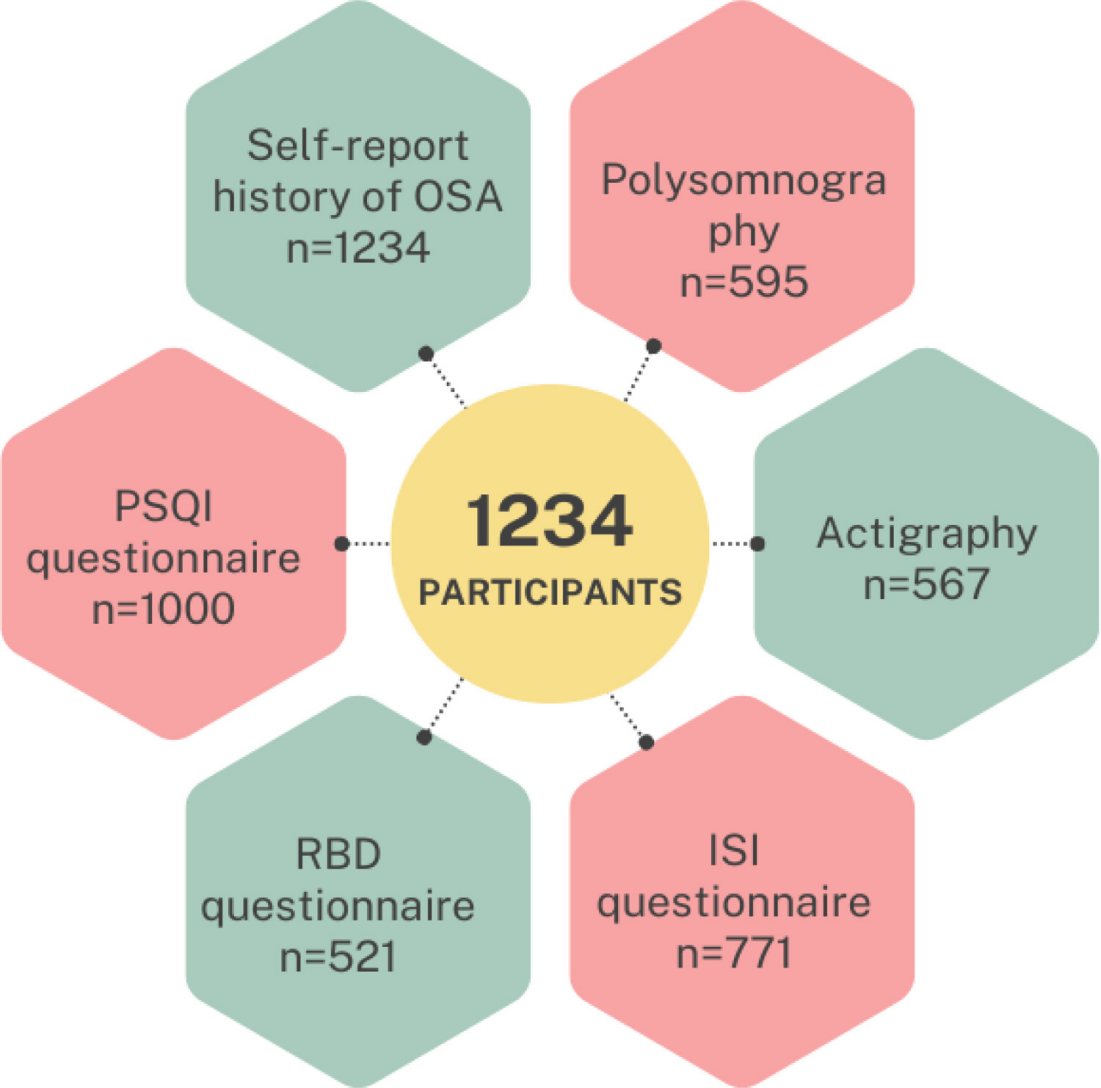
Breakdown of participants, showing sample size for self-reported OSA, polysomnography, actigraphy, ISI questionnaire, PSQI questionnaire, and RBD questionnaire. OSA: obstructive sleep apnea; ISI: insomnia severity index; PSQI: Pittsburgh Sleep Quality Index; RBD: REM sleep behavior disorder.

The final sample (n = 1234) included in this study comprised a variety of sleep-related data sources. This is reflected in Figure 1 below. The demographic and clinical characteristics of the 1234 participants are presented in Table 1. The sample ranged in age from 50 to 89 years (mean age 67.2 ± 9.2) and 46% (563/1234) were male. The sample overall was classified as ‘overweight’ (BMI of 25–29), with low levels of depressive symptoms (GDS-15), good quality of life, low burden of medical diseases (CIRS-G), and within the stage 1 hypertensive range (systolic between 130–139 and diastolic 80–89). In terms of cognition, the mean estimated pre-morbid IQ fell within the ‘average’ range, with relatively intact global cognition on gross screening tools (MMSE). Neuropsychological tests showed that in terms of cognitive classifications, 27% (337) had SCI, 61% met clinical criteria for MCI (including 38% (464/1234) with naMCI and 23% (286/1234) with aMCI; 21% (253/1234) had single-domain MCI and 40% (499/1234) had multi-domain MCI. Twelve percent (147/1234) met the criteria for dementia.

**Table 1.** Demographic and clinical characteristics for whole sample.

Variable	n	mean (SD)
Age, y	1234	67.2 ± 9.2
% male	1234	46% (563/1234)
Education, y	1171	13.7 ± 3.2
Premorbid IQ	1161	105.1 ± 11.4
MMSE	1167	28.0 ± 4.4
Body Mass Index	1135	28.7 ± 11.8
GDS-15	1164	3.8 ± 3.7
WHOQOL	841	3.9 ± 0.9
CIRS-G	1161	4.9 ± 3.6
Alcohol, drinks per week	1172	5.0 ± 7.2
Current major depression, yes	1159	12% (140/1159)
Systolic blood pressure (mmHg)	940	137.2 ± 19.0
Diastolic blood pressure (mmHg)	940	82.2 ± 13.2

MMSE: Mini-Mental State Examination; GDS-15: depressive symptoms as measured by the Geriatric Depression Scale (15 item); WHOQOL: World Health Organization Quality of Life; CIRS-G: Cumulative Illness Rating Scale-Geriatric version.

### Rates of sleep disorders and disturbances

#### Obstructive sleep apnea

**Figure 2.**
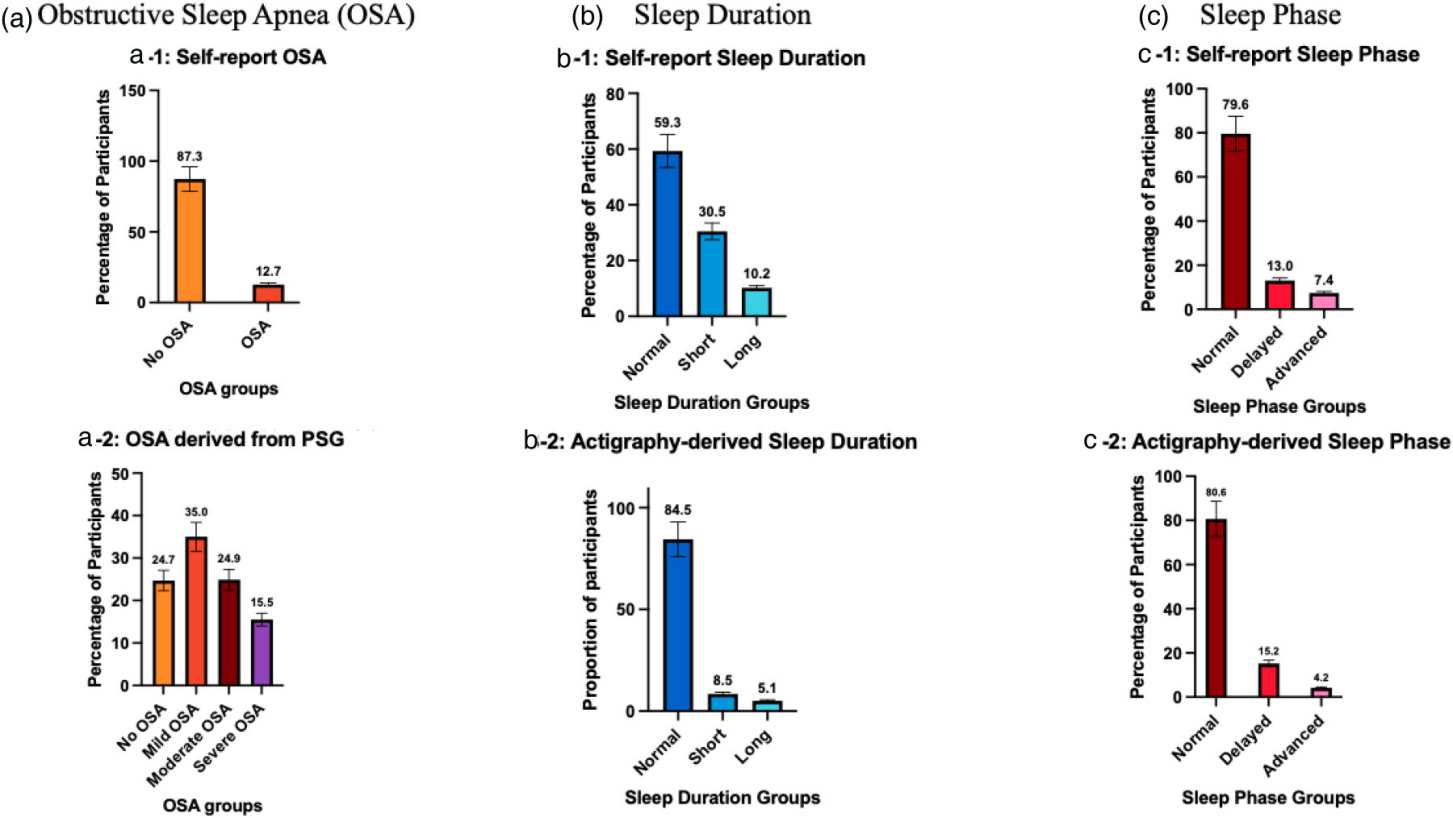
Histograms illustrating the percentage of older adults at-risk of dementia for a) obstructive sleep apnea (OSA), b) short and long sleep duration, and c) delayed and advanced sleep phase. a) For OSA, 12.7% had a prior diagnosis of OSA before attending the memory clinic and upon polysomnography (PSG) 75.3% had OSA. More specifically, 35% have mild, 24.9% have moderate, and 15.5% have severe OSA. b) For long and short sleep durations, self-report revealed 30.5% have short sleep duration (<6hours) and 10.2% have long sleep duration (>9hours). Actigraphy data suggested lower rates of 8.5% with short and 5.1% with long sleep duration. c) For sleep phase, based on self-report 13.0% had delayed sleep phase while 7.4% had advanced sleep phase. Aligned with actigraphy data which revealed 15.2% with delayed and 4.2% with advanced sleep phase.

From self-report during clinician interviews, 12.7% (157/1234) of participants reported they were previously diagnosed with OSA (see Figure 2(a)). Of the participants who reported a previous OSA diagnosis, 35.7% (56/157) reported current CPAP use. Out of 1234 participants, 595 participants completed the optional PSG. Of these, 75.3% (n = 448/595) were classified as having at least mild OSA (AHI ≥5), while 24.7% (n = 147/595) were classified as no OSA (AHI 0–4). Of the total sample, 35.0% (n = 208/595) had mild OSA, 24.9% (n = 148/595) had moderate OSA, and 15.5% (n = 92/595) had severe OSA severity.

#### Short and long sleep duration

Self-report sleep duration derived from PSQI showed that 30.5% (n = 305/1000) of patients had short sleep, 59.3% (n = 593/1000) had normal sleep duration and 10.2% (n = 102/1000) had long sleep duration (see Figure 2(b)). Of those with both PSQI and PSG, 25.9% (n = 125/ 483) participants had both short sleep duration and OSA, while 4.3% (n = 21 / 483) had both long sleep duration and OSA. Actigraphy-derived sleep duration showed lower prevalence, more specifically 8.5% (n = 48/567) had short sleep, 84.5% (n = 479/567) had normal sleep, and 5.1% (n = 29/567) had long sleep duration.

#### Advanced and delayed sleep phase

For the advanced sleep phase, 8.7% (87/1000) met criteria based on self-report while 3.9% (n = 22/567) met criteria based on actigraphy. For the delayed sleep phase, 16.0% (n = 160/1000) met criteria based on self-report which was similar (15.7%, n = 89/567) to that based on actigraphy.

#### Insomnia

**Figure 3.**
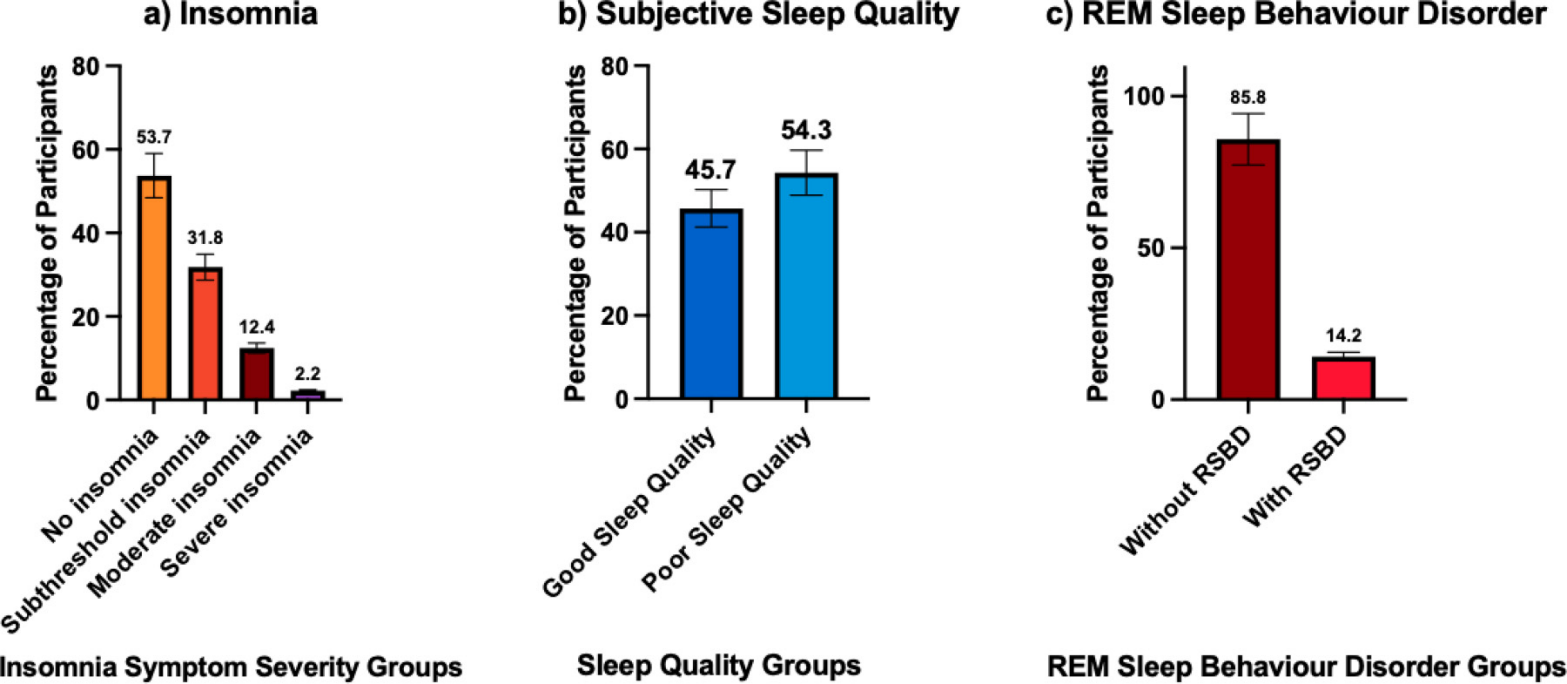
Histograms illustrating percentage of older adults attending memory clinics with a) insomnia symptoms, b) poor sleep quality, and c) REM sleep behavior disorder. a) For insomnia symptoms, 25.7% had subthreshold, 10.6% had moderate, and 1.4% had severe insomnia symptoms. b) For subjective sleep quality, 54.3% had poor sleep quality, c) for REM sleep behavior disorder, 14.2% met cut-off (≥5) on the REM sleep behavior disorder screening questionnaire for REM sleep behavior disorder.

Insomnia symptoms, as captured by the ISI (n = 771), showed that 25.7% (n = 198/771) self-reported subthreshold insomnia, 10.6% (n = 82/771) self-reported moderate clinical insomnia, and 1.4% (n = 11/771) self-reported severe clinical insomnia, while 62.3% (n = 480/771) did not self-report any clinically significant symptoms (see Figure 3(a)).

#### Poor sleepers

In terms of subjective sleep quality, 45.7% (n = 457/1000) of participants were classified as having ‘good’ sleep quality while 54.3% (n = 543/1000) were classified as having poor sleep (see Figure 3(b)).

#### REM behavior disorder risk

For RBD, 14.2% (n = 74/521) of participants screened positive on the REM Sleep Behavior Disorder Screening Questionnaire (see Figure 3(c)).

### Concordance of sleep phase and duration between self-report and actigraphy

A total of 526 participants had both actigraphy and PSQI data. The agreement between self-report and actigraphy in classifying short, normal, and long sleep duration was statistically significant (see Table 2 for cross-tabulations). However, it did not meet our predefined Cohen's Kappa cut-off value of 0.6 (Kappa = 0.135, p = 0.001). Among participants who had short sleep duration on actigraphy, 37.0% overestimated their sleep duration, incorrectly reporting it as either normal or long. Similarly, underestimation of sleep duration was more pronounced among those with long sleep duration on actigraphy, where 73.0% of these individuals self-reported sleep as either short or normal duration. Lastly, among those with normal sleep duration on actigraphy, 38.9% inaccurately self-reported their sleep duration as either short or long.

**Table 2.** Cross-tabulations comparing the classifications of sleep duration derived from self-report and actigraphy.

		Self-report sleep duration#		
		Short	Normal	Long
Actigraphy-derived sleep duration	Short	28	14	3
	Normal	139	282	34
	Long	6	13	7

Number of participants in each category.

# As derived from the PSQI.

The agreement between self-report and actigraphy in classifying advanced, delayed, and normal sleep phases was statistically significant (see Table 3 for cross-tabulations). However, it did not meet our predefined Cohen's Kappa cut-off value of 0.6 (Kappa = 0.373, p = 0.001). Further analysis of individual agreement between self-report and actigraphy sleep phase classifications revealed that for both advanced and delayed sleep phase, the agreement was statistically significant but did not meet our predefined Cohen's Kappa cut-off of 0.6 (Kappa = 0.235, p = 0.001; Kappa = 0.432, p = 0.001 respectively). Among participants with objectively measured advanced phase, 53.0% self-reported either normal or delayed sleep phase. Likewise, 42.7% of those with delayed sleep phase on actigraphy, self-reported as having either normal or advanced sleep phase. Lastly, 10.0% of individuals with normal sleep phase on actigraphy self-reported as having delayed or advanced sleep phase. As an exploratory analysis, we examined the associations between sleep efficiency measured by self-report and actigraphy, as well as wake after sleep onset measured by PSG and actigraphy (see Supplemental Table 1).

**Table 3.** Cross-tabulations of advanced and delayed sleep phases.

		Self-report sleep phase		
		Normal	Advanced	Delayed
Actigraphy-derived sleep phase	Normal	383	22	21
	Advanced	9	7	3
	Delayed	46	0	35

Number of participants in each category.

### Neuropsychological differences in those with and without sleep disorders and disturbance

All group means and standard deviations for each cognitive test are presented in Table 4. When examining group differences in cognition, analyses excluded participants with dementia and controlled for age, sex, and education.

**Table 4.** Group differences in global cognition and neuropsychological testing between those with and without sleep disorders and disturbances.

		MMSE	Processing speed	Verbal memory	Executive function
OSA	Without and mild OSA	28.8 ± 1.9	34.2 ± 13.8	8.7 ± 4.0	79.4 ± 40.4
	Moderate and severe OSA	28.0 ± 3.2	37.5 ± 14.9	7.3 ± 4.3	92.3 ± 50.8
Group differences		p = 0.019, No OSA > OSA	p = 0.390, ND	p = 0.008, No OSA > OSA	p = 0.066, ND

Sleep duration	Short	28.7 ± 1.6	35.7 ± 13.4	8.6 ± 4.1	89.1 ± 43.1
	Normal	28.7 ± 1.9	37.5 ± 18.0	7.8 ± 4.2	92.0 ± 50.2
	Long	27.1 ± 3.2	48.0 ± 34.3	4.6 ± 4.2	132.7 ± 79.6
Group differences		p = 0.001, short = normal > long	p = 0.001, short = normal < long	p = 0.001, short = normal > long	p = 0.001, short = normal < long

Insomnia	No insomnia	28.2 ± 3.5	37.7 ± 18.0	7.7 ± 4.3	79.9 ± 48.3
	Subthreshold insomnia	28.8 ± 1.6	33.9 ± 11.7	8.8 ± 4.3	84.2 ± 36.8
	Moderate -severe insomnia	28.7 ± 2.5	33.1 ± 11.9	9.5 ± 3.4	86.9 ± 43.4
Group differences		p = 0.956, ND	p = 0.273, ND	p = 0.064, ND	p = 0.115, ND

Sleep quality	Good	28.5 ± 2.3	21.9 ± 1.1	7.2 ± 4.4	95.8 ± 56.1
	Poor	28.7 ± 1.7	16.4 ± 0.7	8.3 ± 4.1	93.4 ± 49.5
Group differences		p = 0.212, ND	p = 0.479, ND	p = 0.003, Poor > Good	p = 0.649, ND

ANCOVA were conducted whilst controlling for age, sex, and education and excluded those with dementia. The reported p-value represents the overall effect from the respective sleep disturbance on cognitive outcome after adjusting for covariates. OSA analyses excluded those undergoing continuous positive airway pressure. MMSE: Mini-Mental State Examination; OSA: obstructive sleep apnea; ND: no difference.

Older adults with moderate and severe OSA (measured by polysomnography) had poorer global cognition (Cohen's *d* = 0.30, p < 0.001), and poorer verbal memory (Cohen's *d* = 0.33, p < 0.001) than those without and with mild OSA. These findings continued to be significant even after adjusting for depressive symptoms and the burden of medical diseases (both p < 0.05). There were no differences between groups on processing speed or executive functioning (both p > 0.05).

For subjective sleep duration, those with long sleep duration had poorer neuropsychological performance on all domains including global cognition (MMSE, Cohen's *d* = 0.61, p < 0.001; Cohen's *d* = 0.63, p < 0.001), processing speed (Cohen's *d* = 0.38, p < 0.001; Cohen's *d* = 0.47, p < 0.001), verbal memory (Cohen's *d* = 0.76, p < 0.001; Cohen's *d* = 0.96, p < 0.001), and executive function (Cohen's *d* = 0.61, p < 0.001; Cohen's *d* = 0.68, p < 0.001) compared to those with normal and short sleep duration, respectively. All findings remained significant after further controlling for depressive symptoms and the burden of medical diseases (all p < 0.001). There were no group differences between those with normal and short sleep duration (p > 0.05). As part of an exploratory analysis, we repeated the analysis utilizing objective sleep duration, measured via actigraphy. In summary, MMSE scores were lower in individuals with either short or long sleep duration compared to those with normal sleep duration (p < 0.01). However, there were no other overall group differences on processing speed, verbal memory, and executive function after adjusting for covariates (all p > 0.05). Detailed results are provided in Supplemental Table 5.

For insomnia, individuals with moderate or severe clinical insomnia were classified as the ‘insomnia’ group (n = 93/771). There were no group differences between those with no insomnia, subthreshold insomnia, and insomnia on any neuropsychological test (all p > 0.05).

Surprisingly, older adults with poor sleep quality performed better on verbal memory performance than those with good sleep quality (Cohen's *d* = 0.26, p < 0.001). This was significant after further adjusting for depressive symptoms and medical disease burden (p < 0.001).

For the first sensitivity analysis, 288 participants were taking antidepressants or psychotropic medication. The sensitivity analyses revealed that the associations between OSA, sleep duration, and sleep quality and cognition remained robust even after excluding participants taking antidepressant or psychotropic medication (detailed analyses are provided in Supplemental Table 2).

We conducted sensitivity analyses to explore potential sex differences in the relationship between the sleep disorders and disturbances and cognition. Detailed results are provided in Supplemental Tables 3 and 4. For OSA, the effect on MMSE was significant in females but this did not survive FWER corrections. The effect of OSA on verbal learning in females remained significant, even after corrections. For sleep duration, findings remained the same regardless of sex except for processing speed and executive function in males which were not significant (executive function was significant but did not survive FWER corrections). For insomnia, females with moderate to severe symptoms performed worse than those without insomnia symptoms, though it did not survive FWER corrections. Lastly, regarding sleep quality, females with poor sleep quality demonstrated better MMSE performance. All other results remained the same regardless of sex.

### Sleep disorders and disturbances predict cognitive classification

Lastly, the multinomial logistic regression between subjective sleep duration and cognitive diagnosis (SCI, naMCI, and aMCI) was statistically significant (χ²(4) = 23.104, p < 0.001). More specifically, it demonstrated that those with subjective short and normal sleep duration had lower odds of having aMCI than those with subjective long sleep duration (odds ratio of 0.202, p < 0.001 and odds ratio of 0.256, p < 0.001, respectively). Subjective sleep duration was not predictive of naMCI membership (p > 0.05). No other sleep disturbances predicted either naMCI or aMCI membership (p > 0.05).

## Discussion

This study aimed to characterize various forms of sleep disturbance in a large memory clinic sample. Overall, we found that sleep disturbances are common, particularly OSA, followed by overall complaints of poor sleep quality, which occurred in over half of the sample. Short sleep duration and sub-threshold to full threshold insomnia were also commonly self-reported by around one-third of patients. Self-reported delayed sleep phase and symptoms of RBD occurred in 16% and 14% of patients, whilst subjective long sleep and advanced sleep phase were less commonly reported (5% and 9% respectively). Notably, a key finding of this study was the marked discrepancies between self-reported sleep disturbances and those found using objective measures of either PSG or actigraphy.

It is significant that using gold-standard PSG, three-quarters of patients (75.3%) met AHI criteria for at least mild OSA and 40.4% had at least moderate OSA. This was in marked contrast to only 12.7% of participants reporting a prior diagnosis of OSA. Furthermore, only 35.7% of those with a prior diagnosis were using CPAP therapy. Given the accumulating body of work linking OSA pathophysiology to cognitive decline,^17^ brain structural change,^12,51^ the accumulation of amyloid,^52^ and dementia risk,^19^ the striking underdiagnosis and under-treatment of OSA is a key finding. Furthermore, aligned with prior work, older adults with OSA in this memory clinic setting performed worse on measures of global cognition and verbal learning compared to those with mild or no OSA, after adjusting for age, sex, and education. Furthermore, these findings remained significant after adjusting for depressive symptoms and medical disease burden. Furthermore, our sensitivity analysis suggested that the effect of OSA on cognition was largely driven by females. In order to justify widespread screening in this setting,^53^ a key question remaining for the field is to establish a robust evidence base for the treatment of OSA as a method to slow the cognitive decline leading to dementia.^54^

The examination of subjective sleep duration was also of interest given concerning reports linking short sleep duration with amyloid-β deposition^55–57^ and progression to dementia longitudinally^58^ and long sleep with poorer executive functioning and great amyloid-β burden.^56^ Self-reported abnormal sleep duration was noted in 40.7% of participants, with 30.5% reporting short sleep (<6 h) and 5% reporting long sleep (>9 h). Notably, actigraphy-derived data indicated markedly lower rates of abnormal sleep duration, evident in only 13.6% of participants, with 8.5% having short and 5.1% having long sleep duration. Along this continuum of cognitive decline, our data did not support a relationship between short sleep and cognitive performance. By contrast, subjective long sleepers had poorer global cognition, processing speed, memory, and executive function, as well as higher odds of having aMCI compared to those with normal (odds ratio = 0.202) or short sleep duration (odds ratio = 0.256). This relationship remained significant after adjusting for depressive symptoms and the burden of medical diseases, suggesting that depressive symptoms and other medical diseases do not fully explain the relationship between long sleep duration and cognitive impairment. These findings are aligned with a recent meta-analysis that suggests long sleep duration may be a risk factor for dementia incidence.^59^ Our study extends this evidence by demonstrating prolonged subjective sleep duration is apparent at an earlier clinical stage, specifically in individuals with amnestic MCI. Additionally, our exploratory analyses suggest that sex may moderate the effect of long sleep duration on cognition, particularly on global cognition and processing speed. Taken together, prolonged sleep duration may be a biomarker for the development of amnestic cognitive impairment and, subsequently, AD. We hypothesize that the increased sleep duration may reflect compensatory sleep in response to a decrease in the efficiency of deep sleep (e.g., decreased slow wave activity). Alternatively, it could reflect an increase in frailty which has been linked to both cognitive impairment^60^ and increased sleep duration.^61^ Further studies are now needed to understand the underlying mechanisms and causative nature of sleep duration and dementia risk.^62^

The relationship between circadian rhythm abnormalities and AD is well established, and likely to contribute to ‘sundowning’ in later stages of AD,^63^ which in turn can impact the quality of life for individuals and those around them.^64^ Phase advance is evident even in the MCI stage^65^ and is linked with lower global cognition^66^ and subsequent cognitive decline^67^ in older adults. In this study, phase advance was self-reported in 8.7% of patients, although these figures were only 3.9% according to actigraphy. Rates of delayed sleep phase were almost double (16% using self-report) and concordant with those obtained via actigraphy (15.7%). Delayed sleep phase has been linked with older age^68^ and with increased depressive symptoms.^69^ Therefore, future research is necessary to determine the effects of sleep interventions such as bright light therapy or melatonin on mood, cognition, and behavioral outcomes in older adults with phase shifts.

Moreover, more than one-third (37.7%) of participants reported at least subthreshold insomnia symptoms, with 12% experiencing clinically significant insomnia. In this memory clinic sample, self-reported insomnia was not associated with neuropsychological performance. While this finding is in contrast to some empirical work in older cognitively intact adults,^70,71^ a recent review highlighted mixed findings regarding insomnia and dementia.^8^ Several factors may explain this discrepancy in findings. First, it may reflect differential risk profiles associated with specific insomnia subtypes (e.g., sleep initiation difficulties may confer greater risk than general insomnia symptoms,^72^ or sleep maintenance difficulties).^73^ For instance, one study demonstrated that individuals with the sleep onset subtype performed worse on memory and verbal fluency tasks compared to controls, whereas those with sleep maintenance subtype did not.^74^ Second, previous studies have been largely conducted in community samples and sleep clinic cohorts, which differ from the memory clinic setting studied here. Memory clinic populations typically include individuals with either subjective or objective cognitive impairment, which may alter the relationship between insomnia and cognition. Moreover, the relationship between insomnia and cognition may also be bidirectional or confer the greatest risk in cognitively intact individuals, compared to those with established neurodegenerative disease. Future research is necessary to confirm whether previous findings in insomnia can be generalized to memory clinic settings and explore how different insomnia subtypes may impact cognition in this population.

Self-reported poor sleep quality was reported by more than half of the sample (54.6%) using the PSQI.^75^ These estimates are slightly lower than previous reports^75^ showing 63% of attendees report poor sleep quality. Our prior work has linked poor sleep quality with depressive symptoms, cognition, antidepressant usage, and alcohol consumption.^75^ While other studies have linked poor sleep quality with impaired quality of life,^76^ which is predictive of healthcare expenditure.^77^ Surprisingly, we found that subjective good sleep quality was linked to poorer verbal memory than individuals with poor sleep quality. This contrasted previous studies in the general population and community settings that have linked self-reported poor sleep quality with poorer global cognition^78^ and executive functioning.^79^ However, our finding aligns with a prior community-based study that reported an association between poorer sleep quality and better learning and memory outcomes.^80^ Notably, their study further revealed this relationship was fully explained by AD pathology biomarkers and hippocampal alterations. One possible explanation is that cognitive impairment and decreased meta-cognition impedes the accuracy of self-reported symptoms on the PSQI, as it probes for disturbance over the last month. Given subjective long sleep duration has a moderate effect on verbal memory, an alternative explanation is that the PSQI's calculation of poor sleep quality does not consider long sleep duration as adverse. Thus, individuals with subjective long sleep duration may appear to have more superior self-reported good sleep quality. Future work is necessary to confirm these findings in similar cohorts.

Lastly, 14.2% of this sample endorsed symptoms suggestive of RBD, suggesting underlying synucleinopathy pathology may be present in at least a small subset. These findings are similar to prior studies suggesting that questionnaire screening in clinical settings shows up to 14.4% positive for RBD.^81^ However, upon PSG confirmation, the prevalence of RBD is similar to community samples, which is approximately 2%.^82^ Notably, The RBDSQ has only 56% specificity in patients with other sleep disorders, and the ability of screening questionnaire to predict the lack of atonia during REM sleep, which is a key diagnostic criterion for iRBD, remains low (see review by Bramich et al.).^83^ Nonetheless, screening for probable RBD in the cognition clinic setting is feasible and may inform clinical triage for PSG with EMG as well as case-finding approaches for synucleinopathies, which may be attractive if disease-modifying therapies become available.

This study has highlighted another major challenge that pertains to the optimal measurement of sleep in this setting. Certainly, self-report measures could be considered to be the most feasible for both clinical staff and patients. However, this study has shown poor concordance between self-report and actigraphy-derived measures (notably for sleep duration and sleep phase). It has been postulated that self-reported experiences of sleep may not be reflective of objective measures but rather the microarchitecture of sleep^84^ but this requires empiric examination. The accuracy of self-reporting may also be questionable in those with cognitive impairment.^26,85^ Furthermore, increased discrepancy or misperception of sleep has been linked to impaired executive function, and to amyloid burden,^86^ again raising doubts about using self-report measures in those with cognitive impairment. Collectively, such work, coupled with the current findings, has significant implications for the generalization of studies that rely exclusively on either self-report or objective sleep measures, and it may be crucial to consider both methods to obtain a comprehensive understanding of sleep patterns and their impacts. Further research is warranted to explore the potential of sleep misperception as a biomarker for cognitive decline and dementia risk.

Overall, these study findings are noteworthy for the memory clinic setting, since such individuals have received formal referrals for cognitive concerns, and hence sleep disturbances are not typically their predominant complaint. However, given a possible etiological role of sleep disturbance in dementia onset and progression,^8^ and the critical importance of sleep for daily functioning,^87^ this setting may present an ideal window of opportunity for screening sleep disturbances and for implementing treatments to optimize sleep, and that may in turn, improve cognition.^88^ Given that sedative hypnotics are not recommended for the older age groups and that CPAP presents challenges in adherence, a large hurdle in this field is the dearth of high-quality treatment trials of both newer pharmacological (e.g., orexin-based) and non-pharmacological (e.g., CBT-I, acoustic stimulation) strategies. Indeed, some efforts are underway in this field, and preliminary evidence for older adults with MCI is in its infancy, albeit promising. For instance, in an RCT comprising a small sample of n = 35 (2018)^89^ people with MCI, four sessions of cognitive behavioral therapy for insomnia (CBT-I) was shown to improve subjective sleep quality and reduce daytime sleepiness. The study was not designed nor powered to detect changes in actigraphy measures, depressive symptoms, or cognitive function. In 2022, Hoyos and colleagues^88^ in a pilot randomized controlled trial of 26 participants with both MCI and OSA showed that CPAP treatment for 12-weeks was associated with improvements in verbal learning and memory. Likewise, in preliminary data from the MEMORIES study, Richard et al.^90^ reported that adherence to CPAP (<4 h a night) for 1-year was linked to improvements in processing speed, memory, and attention compared to those who were non-adherent. Whether interventions for sleep can prevent or slow cognitive decline in older memory clinic samples but feasibility trials are underway.^91^ Future trials for this setting would warrant designing in concert with implementation science frameworks to ensure that they are feasible, acceptable, cost-effective and ultimately sustainable.^92^

The current study has some limitations. First, participants who completed PSG tended to report poorer subjective sleep quality and greater insomnia severity (Supplemental Table 6). While our high rates are consistent with rates reported by previous studies of OSA in MCI,^25^ caution must be taken when interpreting the rates of OSA, as there may have been a selection bias towards those with poorer sleep. Second, not all participants completed all assessments. Nonetheless, the current study represents the largest assessment of sleep disturbances in a memory clinic utilizing both gold-standard objective and self-report subjective measures concurrently. Third, our sample is likely to include a more heterogeneous group of people with various underlying neurodegenerative pathologies. We do not have data on Alzheimer's disease biomarkers, which is not atypical for an Australian memory clinic currently. Enriching the sample with these and examining the prognostic value of sleep disturbance in this setting is now warranted and this work is underway. Our study utilized the widely used RBD questionnaire as a screening tool for RBD. However, we did not confirm the accuracy of this questionnaire using video PSG. Prior studies have shown that while up to 14.4% of individuals may screen positive for RBD using questionnaires,^70^ only 2% receive a confirmed diagnosis through video PSG.^82^ This discrepancy may be attributed to the influence of other sleep conditions such as NREM parasomnias, OSA, and periodic leg movements, which can inflate positive screening rates. A further limitation is the absence of screening for restless legs syndrome, a common condition in older adults. Future research is necessary to screen for restless legs syndrome and to better understand its impact on cognition. Finally, although our setting is a memory and cognition clinic, it differs somewhat from typical Australian memory clinics.^21^ Notably, it includes people with concerns about their memory from age 50 onwards, has a lower MMSE limit of 20, and has inclusion and exclusion criteria. Thus, the sample is around a decade younger than many clinics, has higher rates of MCI than dementia,^93^ and less diversity in terms of ethnicity, socioeconomic status, as well as medical, neurological and psychiatric comorbidities, head injury, and substance abuse. This limits the representativeness of our sample and future studies should seek to confirm our findings in the broader memory clinic health setting.

In conclusion, the high prevalence of sleep disorders and disturbances in memory clinic settings underscores the critical need for early screening and intervention, which may significantly benefit cognitive, behavioral, mood and quality of life outcomes. Our findings reinforce the link that sleep apnea and sleep duration are linked to cognitive impairment, while also revealing a poor concordance between objective and subjective sleep measures. Future studies must prioritize the inclusion of both objective and subjective sleep measures to deepen our understanding of sleep's impact on dementia risk and to develop more effective sleep interventions.

## Supplemental Material

sj-docx-1-alz-10.1177_13872877251338065 - Supplemental material for Sleep disturbances and disorders in the memory clinic: Self-report, actigraphy, and polysomnography Supplemental material, sj-docx-1-alz-10.1177_13872877251338065 for Sleep disturbances and disorders in the memory clinic: Self-report, actigraphy, and polysomnography by Aaron Lam, Dexiao Kong, Angela L D’Rozario, Catriona Ireland, Rebekah M Ahmed, Zoe Menczel Schrire, Loren Mowszowski, Johannes Michaelian, Ron R Grunstein and Sharon L Naismith in Journal of Alzheimer's Disease
