# Supplementary material for: Sleep disturbances and disorders in the memory clinic: Self-report, actigraphy, and polysomnography
Source: J Alzheimers Dis. 2025 May 5;106(1):78–93. doi: 10.1177/13872877251338065 (PMC12231846; doi:10.1177/13872877251338065)
Supplement: sj-docx-1-alz-10.1177_13872877251338065 - Supplemental material for Sleep disturbances and disorders in the memory clinic: Self-report, actigraphy, and polysomnography [file sj-docx-1-alz-10.1177_13872877251338065.docx]

**Supplemental Material**

**Sleep disturbances and disorders in the memory clinic: Self-report, actigraphy, and polysomnography**

**Supplemental Table 1.** Comparison of sleep efficiency and wake after sleep onset across different modalities

| PSQI versus Actigraphy | | | |
| --- | --- | --- | --- |
|  | Spearman’s rho | p |  |
| Sleep efficiency | 0.097 | 0.024 |  |
| PSG versus Actigraphy | | | |
|  | Spearman’s rho | p |  |
| Wake after sleep onset | 0.352 | 0.001 |  |

PSQI: Pittsburgh Sleep Quality Index; PSG: polysomnography

**Supplemental Table 2.** Sensitivity analysis to examine the group differences in global cognition and neuropsychological testing between those with and without sleep disorders and disturbances without individuals taking antidepressants and psychotropics.

|  |  | | | | MMSE | | | | | | | Processing speed | | | | Verbal memory | | | | | | Executive function | | | |  |
| --- | --- | --- | --- | --- | --- | --- | --- | --- | --- | --- | --- | --- | --- | --- | --- | --- | --- | --- | --- | --- | --- | --- | --- | --- | --- | --- |
| OSA | Without and mild OSA | | | | 28.4 ± 2.4 | | | | | | | 36.7 ± 13.8 | | | | 8.0 ± 4.4 | | | | | | 82.3 ± 47.2 | | | |  |
|  | Moderate and severe OSA | | | | 27.5 ± 3.6 | | | | | | | 40.2 ± 21.2 | | | | 6.6 ± 4.5 | | | | | | 89.2 ± 51.9 | | | |  |
| Group differences | | | | | p=0.288, ND | p=0.945, ND | | | | | | | | | | | p=0.003, No OSA > OSA | | | | | | | p=0.613, ND | |  |
|  | | MMSE | | | | | | | | Processing speed | | | | | | | | Verbal memory | | | | | | | Executive function |  |
| Sleep duration | Short | | | | 28.7 ± 1.6 | | | | | | | 35.5 ± 14.3 | | | | 8.6 ± 4.1 | | | | | | 88.5 ± 46.3 | | | |  |
|  | Normal | | | | 28.7 ± 1.9 | | | | | | | 36.3 ± 17.6 | | | | 7.9 ± 4.1 | | | | | | 87.4 ± 45.5 | | | |  |
|  | Long | | | | 27.0 ± 2.3 | | | | | | | 51.3 ± 36.3 | | | | 3.4 ± 3.6 | | | | | | 141.2 ± 81.8 | | | |  |
| Group differences | | | | | p=0.001, Short = normal > Long | | | | p=0.001, Short = normal < Long | | | | | | | | p=0.001, Short = normal > Long | | | | | | p=0.001, Short = normal < Long | | |  |
|  | | MMSE | | | | | Processing speed | | | | | | Verbal memory | | | | | | Executive function | | | | | | |  |
| Insomnia | No insomnia | | | | 28.1 ± 3.7 | | | | | | | 37.5 ± 18.5 | | | | 7.7 ± 4.3 | | | | | | 78.0 ± 49.4 | | | |  |
|  | Subthreshold insomnia | | | | 28.9 ± 1.6 | | | | | | | 33.7 ± 11.9 | | | | 8.5 ± 4.4 | | | | | | 84.8 ± 37.0 | | | |  |
|  | Moderate -severe insomnia | | | | 28.8 ± 1.4 | | | | | | | 33.8 ± 13.2 | | | | 9.2 ± 3.3 | | | | | | 89.3 ± 49.3 | | | |  |
| Group differences | | | p=0.980, ND | | | | | p=0.434, ND | | | | | | p=0.228, ND | | | | | | p=0.291, ND | | | | | |  |
|  | | MMSE | | | | | Processing speed | | | | | | Verbal memory | | | | | | Executive function | | | | | | |  |
| Sleep quality | Good | | | | 28.6 ± 2.1 | | | | | | | 38.3 ± 21.9 | | | | 7.2 ± 4.2 | | | | | | 93.0 ± 53.5 | | | |  |
|  | Poor | | | | 28.7 ± 1.6 | | | | | | | 36.5 ± 15.9 | | | | 8.4 ± 4.1 | | | | | | 90.0 ± 47.9 | | | |  |
| Group differences | | | | p=0.751, ND | | | | | | | p=0.057, ND | | | | p=0.001, Poor > Good | | | | | | p=0.372, ND | | | | | |
| ANCOVA were conducted with adjustment to age, sex, education level, depression symptoms, and medical disease burden and excluded those with dementia. The reported p-value represents the overall effect from the respective sleep disturbance on cognitive outcome after adjusting for covariates. OSA analyses excluded those undergoing continuous positive airway pressure. MMSE: Mini-Mental State Examination; OSA: obstructive sleep apnoea; ND: no difference. | | | | | | | | | | | | | | | | | | | | | | | | | |  |

**Supplemental Table 3.** Sensitivity analysis to examine sex differences – Between group global cognition and neuropsychological testing between those with and without sleep disorders and disturbances in male subjects

|  |  | MMSE | Processing speed | Verbal memory | Executive function |  |
| --- | --- | --- | --- | --- | --- | --- |
| OSA | Without and mild OSA | 28.8 ± 1.7 | 34.8 ± 11.7 | 7.9 ± 4.1 | 81.5 ± 45.3 |  |
|  | Moderate and severe OSA | 28.2 ± 2.2 | 37.4 ± 15.7 | 6.3 ± 3.7 | 94.5 ± 48.2 |  |
| Group differences | | p=0.561, ND | p=0.977, ND | p=0.133, ND | p=0.798, ND |  |
|  | | MMSE | Processing speed | Verbal memory | Executive function |  |
| Sleep duration | Short | 28.7 ± 1.6 | 35.5 ± 14.3 | 8.6 ± 4.1 | 88.5 ± 46.3 |  |
|  | Normal | 28.7 ± 1.9 | 36.3 ± 17.6 | 7.9 ± 4.1 | 87.4 ± 45.5 |  |
|  | Long | 27.0 ± 2.3 | 51.3 ± 36.3 | 3.4 ± 3.6 | 141.2 ± 81.8 |  |
| Group differences | | p=0.010, short = normal > Long | p=0.403, ND | p=0.001, short = normal > Long | p=0.039, ND |  |
|  | | MMSE | Processing speed | Verbal memory | Executive function |  |
| Insomnia | No insomnia | 28.6 ± 3.5 | 37.9 ± 17.8 | 7.1 ± 4.1 | 78.0 ± 49.4 |  |
|  | Subthreshold insomnia | 28.6 ± 1.9 | 33.1 ± 9.7 | 8.0 ± 3.8 | 84.8 ± 37.0 |  |
|  | Moderate -severe insomnia | 29.0 ± 1.4 | 33.8 ± 13.4 | 9.0 ± 3.8 | 89.3 ± 49.3 |  |
| Group differences | | p=0.724, ND | p=0.318, ND | p=0.530, ND | p=0.504, ND |  |
|  | | MMSE | Processing speed | Verbal memory | Executive function |  |
| Sleep quality | Good | 28.6 ± 1.8 | 38.3 ± 21.9 | 7.2 ± 4.2 | 93.0 ± 53.5 |  |
|  | Poor | 28.5 ± 1.6 | 36.5 ± 15.9 | 8.4 ± 4.1 | 90.0 ± 47.9 |  |
| Group differences | | p=0.392, ND | p=0.720, ND | p=0.001, Poor > Good | p=0.928, ND |  |
| ANCOVA were conducted with adjustment to age, education level, depression symptoms, and medical disease burden and excluded those with dementia. The reported p-value represents the overall effect from the respective sleep disturbance on cognitive outcome after adjusting for covariates. OSA analyses excluded those undergoing continuous positive airway pressure. MMSE: Mini-Mental State Examination; OSA: obstructive sleep apnoea; ND: no difference. | | | | | | |

**Supplemental Table 4.** Sensitivity analysis to examine sex differences – Between group global cognition and neuropsychological testing between those with and without sleep disorders and disturbances in female subjects

|  |  | MMSE | Processing speed | Verbal memory | Executive function |  |
| --- | --- | --- | --- | --- | --- | --- |
| OSA | Without and mild OSA | 29.1 ± 1.3 | 33.2 ± 12.6 | 9.8 ± 3.5 | 79.9 ± 32.2 |  |
|  | Moderate and severe OSA | 28.5 ± 2.6 | 34.6 ± 13.2 | 8.2 ± 4.4 | 88.3± 41.0 |  |
| Group differences | | p=0.033, ND | p=0.540, ND | p=0.014, No OSA > OSA | p=0.175, ND |  |
|  | | MMSE | Processing speed | Verbal memory | Executive function |  |
| Sleep duration | Short | 28.7 ± 1.6 | 35.5 ± 14.3 | 8.6 ± 4.1 | 88.5 ± 46.3 |  |
|  | Normal | 28.7 ± 1.9 | 36.3 ± 17.6 | 7.9 ± 4.1 | 87.4 ± 45.5 |  |
|  | Long | 27.0 ± 2.3 | 51.3 ± 36.3 | 3.4 ± 3.6 | 141.2 ± 81.8 |  |
| Group differences | | p=0.001, short = normal > long | p=0.001, short = normal < long | p=0.003, short = normal > long | p=0.003, short = normal > long |  |
|  | | MMSE | Processing speed | Verbal memory | Executive function |  |
| Insomnia | No insomnia | 28.9 ± 2.0 | 36.8 ± 18.4 | 8.6 ± 4.0 | 82.6 ± 43.1 |  |
|  | Subthreshold insomnia | 28.9 ± 1.5 | 34.1 ± 12.6 | 9.5 ± 4.3 | 84.8 ± 38.5 |  |
|  | Moderate -severe insomnia | 28.5 ± 3.1 | 31.2 ± 10.1 | 10.1 ± 3.1 | 90.4 ± 39.0 |  |
| Group differences | | p=0.869, ND | p=0.071, ND | p=0.029, ND | p=0.673, ND |  |
|  | | MMSE | Processing speed | Verbal memory | Executive function |  |
| Sleep quality | Good | 28.5 ± 2.4 | 38.3 ± 21.9 | 7.2 ± 4.2 | 93.0 ± 53.5 |  |
|  | Poor | 28.9 ± 1.5 | 36.5 ± 15.9 | 8.4 ± 4.1 | 90.0 ± 47.9 |  |
| Group differences | | p=0.003, Poor > Good | p=0.044, ND | p=0.008, Poor > Good | p=0.135, ND |  |
| ANCOVA were conducted with adjustment to age, education level, depression symptoms, and medical disease burden and excluded those with dementia. The reported p-value represents the overall effect from the respective sleep disturbance on cognitive outcome after adjusting for covariates. OSA analyses excluded those undergoing continuous positive airway pressure. MMSE: Mini-Mental State Examination; OSA: obstructive sleep apnoea; ND: no difference. | | | | | | |

| **Supplemental Table 5.** Between group differences in global cognition and neuropsychological testing between those with short and long sleep duration vs. normal sleep duration | | | | | | | |
| --- | --- | --- | --- | --- | --- | --- | --- |
|  |  | | MMSE | Processing speed | Verbal memory | | Executive function |
| Sleep duration | Short | | 28.0 ± 3.5 | 36.2 ± 11.8 | 8.9 ± 4.6 | | 87.7 ± 40.3 |
|  | Normal | | 28.9 ± 1.4 | 34.8 ± 13.0 | 8.5 ± 4.0 | | 87.2 ± 44.9 |
|  | Long | | 27.7 ± 1.8 | 36.0 ± 11.2 | 6.9 ± 3.6 | | 103.3 ± 48.1 |
| Group differences | | p=0.005, Normal > Short = Long | | p=0.860, ND | p=0.415, ND | p=0.744, ND | |
| ANCOVA were conducted with adjustment to age, sex, education level, and depression symptoms and excluded those with dementia. The reported p-value represents the overall effect from sleep duration on cognitive outcome after adjusting for covariates. The overall ANCOVA p-value is reported. OSA analyses excluded those undergoing continuous positive airway pressure. MMSE: Mini-Mental State Examination; OSA: obstructive sleep apnoea; ND: no difference. | | | | | | | |

**Supplemental Table 6.** Comparing demographics of participants who completed PSG and those who did not

|  | Did not complete PSG  n=639 | Completed PSG  n=595 | Test statistic | p | |
| --- | --- | --- | --- | --- | --- |
| Age, y^a^ | 67.4 ± 9.5 | 67.0 ± 8.4 | 0.8 | 0.430 | |
| Sex, male^b^ | 44% (279/639) | 48% (284/595) | 2.1 | 0.152 | |
| Education, years | 13.5 ± 3.2 | 14.0 ± 3.1 | 5.3 | 0.021* | |
| Premorbid IQ | 104.9 ± 10.0 | 105.9 ± 9.3 | 4.5 | 0.034* | |
| MMSE^c^ | 27.6 ± 3.4 | 28.1 ± 2.9 | 13.9 | 0.001** | |
| SCI^b,d^ | 24% (154/639) | 31% (183/595) |  |  | |
| naMCI^b,d^ | 38% (241/639) | 37% (223/595) |  |  | |
| aMCI^b,d^ | 23% (146/639) | 24% (140/595) |  |  | |
| Dementia^b,d^ | 15% (98/639) | 8% (49/595) | 18.1 | 0.001** | |
| CIRS-G^c^ | 4.9 ± 3.6 | 4.9 ± 3.6 | 0.2 | 0.669 | |
| Current Major Depression, yes | 11% (67/605) | 13% (73/554) | 1.2 | 0.273 | |
| GDS-15^c^ | 4.1 ± 3.8 | 3.5 ± 3.6 | 2.1 | 0.149 | |
| Body Mass Index | 26.7 ± 5.1 | 27.2 ± 5.2 | 2.3 | 0.130 | |
| Alcohol use, standard drink/wk | 5.0 ± 7.2 | 5.0 ± 7.2 | 0.1 | 0.797 | |
| Subjective sleep quality | 6.4 ± 4.0 | 7.0 ± 3.9 | 7.8 | 0.005** | |
| Insomnia severity Index^c^ | 5.0 ± 5.4 | 7.6 ± 6.4 | 36.3 | 0.001** | |
| *p<0.05, **p<0.01  Mean and SD are presented unless stated otherwise. ANCOVA conducted adjusting for age and sex unless stated otherwise.  ^a^Independent student t-test.  ^b^Count (percentage) are presented and chi-square goodness of fit is conducted.  ^c^Raw mean and SD is displayed, however group comparison was conducted on the log transformed variable.  ^d^Z-test for independent proportions with Bonferroni p-value correction. Dementia < SCI, naMCI, aMCI for both groups.  MMSE: Mini-Mental State Examination; SCI: subjective cognitive impairment; aMCI: amnestic mild cognitive impairment; naMCI: non-amnestic mild cognitive impairment; GDS-15: Geriatric Depression Scale 15-item; PSQI: Pittsburg Sleep Quality Index.  Education data missing for 22 no PSG and 41 PSG participants.  Premorbid IQ data missing for 40 no PSG and 38 PSG participants.  MMSE data missing for 36 no PSG and 38 PSG participants.  CIRS-G data missing for 35 no PSG and 38 PSG participants.  GDS data missing for 37 no PSG and 65 PSG participants.  BMI data missing for 76 no PSG and 56 PSG participants.  Alcohol data missing for 29 no PSG and 33 PSG participants.  Subjective sleep quality data missing for 40 no PSG and 69 PSG participants.  Insomnia Severity Index data missing for 315 no PSG and 148 PSG participants. | | | | |  |
